# MIMO Fuzzy Sliding Mode Control for Three-Axis Inertially Stabilized Platform

**DOI:** 10.3390/s19071658

**Published:** 2019-04-06

**Authors:** Zhanmin Zhou, Bao Zhang, Dapeng Mao

**Affiliations:** 1 Changchun Institute of Optics, Fine Mechanics and Physics, Chinese Academy of Sciences, Changchun 130033, China; zhangb@ciomp.ac.cn (B.Z.); mdp_ciomp@126.com (D.M.); 2 University of Chinese Academy of Sciences, No. 19, Yuquan Rd., Beijing 100049, China

**Keywords:** three-axis inertially stabilized platform, MIMO, sliding mode, fuzzy logic

## Abstract

In this paper, a MIMO (Multi-Input Multi-Output) fuzzy sliding mode control method is proposed for a three-axis inertially stabilized platform. This method is based on the MIMO coupling model of the three-axis inertially stabilized platform in which the dynamic coupling among the three frames, namely the azimuth frame, the pitch frame and the roll frame, is fully considered. Firstly, the dynamic equation of the three-axis inertially stabilized platform is analyzed and its linearized model is obtained. After this, the controller is designed based on the model, during which fuzzy logic is introduced to deal with the frame coupling and the adaptive fuzzy coupling compensation factor is designed to be part of the algorithm. A complete proof of the stability and convergence is also provided in this paper. Finally, the performance of the platform with a MIMO fuzzy sliding mode controller and PI controller is analyzed. The simulation results show that the proposed scheme can guarantee tracking accuracy and effectively suppress the coupling interference between the three frames.

## 1. Introduction

As a very important UAV mission load, the inertially stabilized platform is widely used in the fields of aerial reconnaissance, target indication and positioning, strike calibration, battlefield damage assessment, aerial surveying and mapping [1]. An inertially stabilized platform with good performance can effectively isolate the disturbances occurring in the aircraft in addition to establishing a stable spatial orientation for the optical load’s line of sight and the stable tracking of the designated target. However, due to internal and external disturbances, such as carrier disturbance, friction, mass imbalance, airflow disturbance, output torque fluctuation, engine vibration and the complex frame structure and coupling relationship, it is not easy to achieve high performance control of the system [2,3,4,5].

In engineering applications, the most widely used controllers are still traditional linear controllers, such as PID and lead-lag [6,7,8]. This type of controller has several advantages, being relatively convenient and easy to use. Additionally, they are relatively mature due to years of development and improvement of the applications on the inertially stabilized platform and can achieve good results. However, when it is necessary to further improve the system performance, such controllers have limitations. Firstly, the traditional linear controllers have limited ability to control the various internal and external disturbances and non-linear effects on the inertially stabilized platform and the controller. This depends on whether the accuracy models fully consider the effects of unmodeled dynamics and system parameter changes or not. Secondly, in traditional applications, the control effect of the single control of each frame is not ideal for MIMO systems that have coupling across multiple degrees of freedom [9,10,11].

In theory, the sliding mode can be designed according to the current requirements and it has nothing to do with the disturbance in the system and the change or perturbation of the system parameters. That is to say, the design of this sliding mode is invariant to the disturbance [12,13]. Therefore, sliding mode variable structure control is very suitable for an inertially stabilized platform with complex working conditions and it has been a hot topic in academic research. However, the problem of chattering exists in sliding mode control, which should be focused on in practical applications [14,15,16]. A previous study [17] used a saturated function instead of a switching function to form the basic boundary layer, which effectively weakens the chattering effect. In reference [18], an adaptive adjustment is made to the boundary layer thickness, which is further combined with fuzzy rules to achieve a better buffeting weakening effect. In order to solve the problem of the high switching gain in sliding mode control law being able to easily cause the chattering effect of the system, a sliding mode control based on a disturbance observer is designed for the servo system with strong disturbance in reference [19]. By the feedforward compensation of the disturbance observer, the switching gain of sliding mode control law is effectively reduced and the chattering effect of the system is weakened.

In addition to deal with a complex and large disturbance working environment, this paper designs a MIMO fuzzy sliding mode control method for the three-axis inertially stabilized platform with consideration of the incomplete decoupling and unmodeled coupling between the multiple degrees of freedom that is caused by the separate control of each frame in traditional applications. The algorithm fully considers the dynamic coupling among the three frames of the research object. The main feature of the method is that the chattering effect of sliding mode control can be reduced while the coupling compensation is carried out using fuzzy logic [20,21,22].

This paper is organized as follows: in Section 2, we establish the dynamic model of the three-axis inertially stabilized platform and obtain its linear form. In Section 3, the MIMO fuzzy sliding mode control method is designed based on the model and a complete analysis is provided. A series of simulations validate the effectiveness of the controller in Section 4. Finally, our conclusions are presented in Section 5.

## 2. Dynamic Model of a Three-Axis Inertially Stabilized Platform

**Figure 1:**
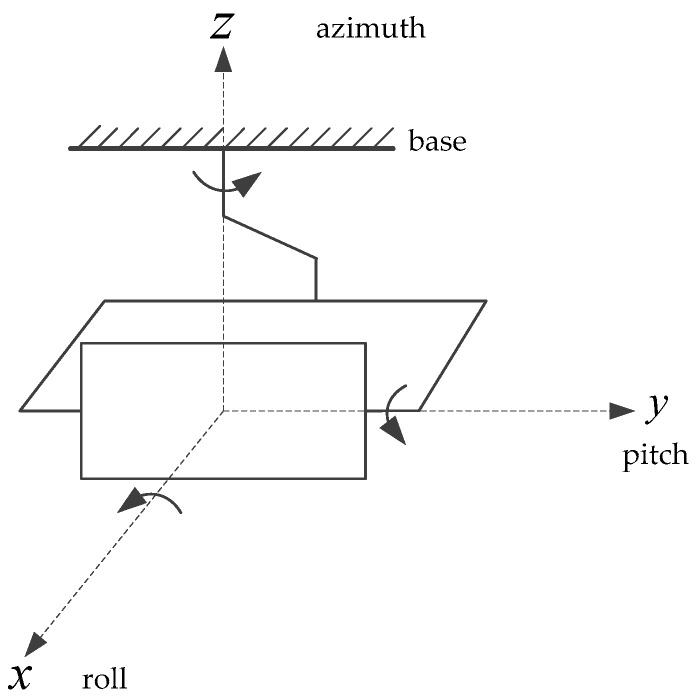
Three-axis airborne inertially stabilized platform.

The research object of this paper is a three-axis inertially stabilized platform. As shown in Figure 1, from the outside to the inside, they are respectively the azimuth frame, roll frame and pitch frame. This platform is essentially created by inserting a roll frame into the two-frame platform of azimuth and pitch.

The three-axis inertially stabilized platform is a system with strong coupling [23,24,25]. Before the establishment of the mathematical model, we need to make the following assumptions:

(1) Ignoring the elastic deformation of frame structures, each frame structure is analyzed according to the rigid body.
(2) The rotation axes of each frame intersect in space and the two adjacent frame shafts are strictly orthogonal.

Four coordinate systems need to be established:

(1) The base coordinate system $Ox_{B}y_{B}z_{B}$ (also known as the UAV coordinate system), which is fixedly connected with the base;
(2) The azimuth frame coordinate system $Ox_{A}y_{A}z_{A}$, which is fixedly connected with the azimuth frame.
(3) The rolling frame coordinate system $Ox_{R}y_{R}z_{R}$, which is fixedly connected with the rolling frame.
(4) The pitching frame coordinate system $Ox_{E}y_{E}z_{E}$, which is fixedly connected to the pitching frame.

**Figure 2:**
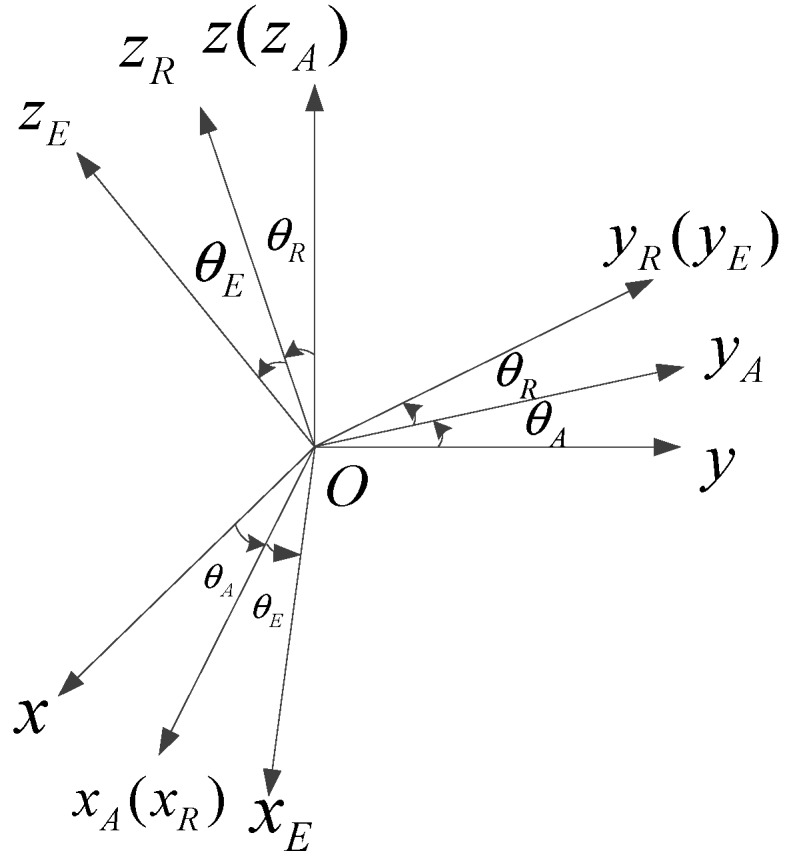
Three-axis coordinate transformation relationship.

The rotation angle of the azimuth frame relative to the base is defined as the azimuth angle $\theta_{A}$; the rotation angle of the rolling frame relative to the azimuth frame is the rolling angle $\theta_{R}$; and the rotation angle of the pitching frame relative to the rolling frame is the pitching angle $\theta_{E}$. The coordinate transformation relationship is shown in Figure 2.

The positive direction of the specified angle follows the right-hand rule. The transformation matrices can be expressed as follows:

(1) $$R_{BA}=\left[\begin{array}{ccc} \cos\theta_{A} & \sin\theta_{A} & 0\\ -\sin\theta_{A} & \cos\theta_{A} & 0\\ 0 & 0 & 1 \end{array}\right],R_{AR}=\left[\begin{array}{ccc} 1 & 0 & 0\\ 0 & \cos\theta_{R} & \sin\theta_{R}\\ 0 & -\sin\theta_{R} & \cos\theta_{R} \end{array}\right],R_{RE}=\left[\begin{array}{ccc} \cos\theta_{E} & 0 & -\sin\theta_{E}\\ 0 & 1 & 0\\ \sin\theta_{E} & 0 & \cos\theta_{E} \end{array}\right]$$

where $R_{BA}$ is the transformation matrix from the base to azimuth frame; $R_{AR}$ is the transformation matrix from the azimuth frame to the roll frame; and $R_{RE}$ is the transformation matrix from the roll frame to the pitch frame. 

The angular velocities of the base, azimuth frame, roll frame and pitch frame in a relative inertia space are defined respectively as $\omega_{B}$, $\omega_{A}$, $\omega_{R}$ and $\omega_{E}$. They can be written in the vector form as follows:

(2) $$\omega_{B}=\left[\begin{array}{c} \omega_{Bx}\\ \omega_{By}\\ \omega_{Bz} \end{array}\right],\omega_{A}=\left[\begin{array}{c} \omega_{Ax}\\ \omega_{Ay}\\ \omega_{Az} \end{array}\right],\omega_{R}=\left[\begin{array}{c} \omega_{Rx}\\ \omega_{Ry}\\ \omega_{Rz} \end{array}\right],\omega_{E}=\left[\begin{array}{c} \omega_{Ex}\\ \omega_{Ey}\\ \omega_{Ez} \end{array}\right]$$

According to the rotation relation, the following relations can be obtained between $\omega_{B}$, $\omega_{A}$, $\omega_{R}$ and $\omega_{E}$:

(3) $$\omega_{A}=R_{BA}\omega_{B}+\left[\begin{array}{c} 0\\ 0\\ {\dot{\theta }}_{A} \end{array}\right],\omega_{R}=R_{AR}\omega_{A}+\left[\begin{array}{c} {\dot{\theta }}_{R}\\ 0\\ 0 \end{array}\right],\omega_{E}=R_{RE}\omega_{R}+\left[\begin{array}{c} 0\\ {\dot{\theta }}_{E}\\ 0 \end{array}\right]$$

The rotational inertia of the three frames of the inertially stabilized platform, namely the azimuth frame, the roll frame and the pitch frame, are respectively defined as $J_{A}=diag\left\{\begin{array}{ccc} J_{Axx} & J_{Ayy} & J_{Azz} \end{array}\right\}$, $J_{R}=diag\left\{\begin{array}{ccc} J_{Rxx} & J_{Ryy} & J_{Rzz} \end{array}\right\}$ and $J_{E}=diag\left\{\begin{array}{ccc} J_{Exx} & J_{Eyy} & J_{Ezz} \end{array}\right\}$ while all the torques acting on three shafts (i.e., the azimuth, pitch and roll shafts) are defined as $T_{A}={\left[\begin{array}{ccc} T_{Ax} & T_{Ay} & T_{Az} \end{array}\right]}^{T}$, $T_{R}={\left[\begin{array}{ccc} T_{Rx} & T_{Ry} & T_{Rz} \end{array}\right]}^{T}$, $T_{E}={\left[\begin{array}{ccc} T_{Ex} & T_{Ey} & T_{Ez} \end{array}\right]}^{T}$, respectively.

According to the rigid body dynamics equation, the elevation frame equation of the three-axis inertially stabilized platform can be obtained as follows:

(4) $$J_{E}{\dot{\omega }}_{E}+\omega_{E}\times J_{E}\omega_{E}=T_{E}$$

where $T_{E}$ can be expanded as:

(5) $$T_{E}=\left[\begin{array}{c} T_{Ex}\\ T_{Ey}\\ T_{Ez} \end{array}\right]=\left[\begin{array}{c} T_{Ex}\\ T_{Edrive}-T_{Efric}\\ T_{Ez} \end{array}\right]$$

where $T_{Ex}$ and $T_{Ez}$ are the reaction torques acting on the pitch frame; $T_{Edrive}$ is the driving torque of the pitching frame motor; and $T_{Efric}$ is the friction torque. Considering the control accuracy, cost and miniaturization, the Permanent Magnet Synchronous Motor (PMSM) is used to drive and control the three frame axes of the three-axis inertially stabilized platform. The current loop is used in the inner loop of the control loop and the bandwidth of the current loop is high enough that the output torque of the motor can be modeled as a proportional function of the control current. 

The effect of friction on a system is very complex. Thus, in order to simplify the analysis, the friction torque $T_{Efric}$ is divided into linear and nonlinear parts. These parts are expressed as follows:

(6) $$T_{Efric}=T_{Ef0}+K_{Ef}{\dot{\theta }}_{E}$$

By expanding the second line of Equation (4) and introducing Equations (5) and (6), the dynamic equation of the pitching frame can be obtained.

(7) $$J_{Eyy}{\dot{\omega }}_{Ey}+\omega_{Ex}\omega_{Ez}(J_{Exx}-J_{Ezz})=T_{Edrive}-T_{Ef0}-K_{Ef}{\dot{\theta }}_{E}$$

In addition, according to Equations (1) and (3), we can obtain the following:

(8) $${\dot{\theta }}_{E}=\omega_{Ey}+\omega_{Bx}\sin\theta_{A}\cos\theta_{R}-\omega_{By}\cos\theta_{A}\cos\theta_{R}-\omega_{Bz}\sin\theta_{R}-{\dot{\theta }}_{A}\sin\theta_{R}$$

From Equations (7) and (8), we can obtain the following:

(9) $$\begin{array}{l} J_{Eyy}{\dot{\omega }}_{Ey}+K_{Ef}\omega_{Ey}+\omega_{Ex}\omega_{Ez}(J_{Exx}-J_{Ezz})=T_{Edrive}-T_{Ef0}\\ -K_{Ef}(\omega_{Bx}\sin\theta_{A}\cos\theta_{R}-\omega_{By}\cos\theta_{A}\cos\theta_{R}-\omega_{Bz}\sin\theta_{R}-{\dot{\theta }}_{A}\sin\theta_{R}) \end{array}$$

According to the analysis of the system, we know that the controlled targets of the three-axis inertially stabilized platform are the angular velocities ${\dot{\omega }}_{Ex}$, ${\dot{\omega }}_{Ey}$ and ${\dot{\omega }}_{Ez}$ of the pitching frame (the innermost frame). The driving torques are the driving torques of the motor on the pitching axis, the roll axis and the azimuth axis. The disturbance comes from the angular velocities $\omega_{Bx}$, $\omega_{By}$ and $\omega_{Bz}$ of the carrier and the disturbance of friction on the system shafting. Similar to the pitch frame, the dynamic equation of the rolling frame and the azimuth frame can be expressed as:

(10) $$J_{R}{\dot{\omega }}_{R}+\omega_{R}\times J_{R}\omega_{R}=T_{R}+T_{RD}$$

(11) $$J_{A}{\dot{\omega }}_{A}+\omega_{A}\times J_{A}\omega_{A}=T_{A}+T_{AD}$$

Similarly, $T_{R}$ and $T_{A}$ can be expanded as follows:

(12) $$T_{R}=\left[\begin{array}{c} T_{Rx}\\ T_{Ry}\\ T_{Rz} \end{array}\right]=\left[\begin{array}{c} T_{Rdrive}-T_{Rfric}\\ T_{Ry}\\ T_{Rz} \end{array}\right],T_{A}=\left[\begin{array}{c} T_{Ax}\\ T_{Ay}\\ T_{Az} \end{array}\right]=\left[\begin{array}{c} T_{Ax}\\ T_{Ay}\\ T_{Adrive}-T_{Afric} \end{array}\right]$$

It is important to note the disturbances $T_{RD}={\left[\begin{array}{ccc} T_{RDx} & T_{RDy} & T_{RDz} \end{array}\right]}^{T}$ and $T_{AD}={\left[\begin{array}{ccc} T_{ADx} & T_{ADy} & T_{ADz} \end{array}\right]}^{T}$ The former represents the projection of the torque acting on the pitch frame on the rolling frame, while the latter represents the projection of the torque acting on the pitch frame and the rolling frame on the azimuth frame.

After this, the first and third lines of Equations (10) and (11) are expanded, respectively. By derivation, the dynamic equations of the rolling frame and azimuth frame can also be obtained:

(13) $$\begin{array}{l} (J_{Rxx}\cos\theta_{E}){\dot{\omega }}_{Ex}+[{\dot{\theta }}_{E}\sin\theta_{E}\left(J_{Rzz}-J_{Ryy}-1\right)+K_{Rf}\cos\theta_{E}]\omega_{Ex}\\ +(J_{Rxx}\sin\theta_{E}){\dot{\omega }}_{Ez}+[J_{Rxx}{\dot{\theta }}_{E}\cos\theta_{E}-{\dot{\theta }}_{E}\cos\theta_{E}(J_{Rzz}-J_{Ryy})+K_{Rf}\sin\theta_{E}]\omega_{Ez}\\ -\sin\theta_{E}(J_{Rzz}-J_{Ryy})\omega_{Ex}\omega_{Ey}+\cos\theta_{E}(J_{Rzz}-J_{Ryy})\omega_{Ey}\omega_{Ez}\\ =T_{RDx}+T_{Rdrive}-T_{Rf0}+K_{Rf}(\omega_{Bx}\cos\theta_{A}+\omega_{By}\sin\theta_{A}) \end{array}$$

(14) $$\begin{array}{l} (-J_{Azz}\sin\theta_{E}\cos\theta_{R}){\dot{\omega }}_{Ex}+(-J_{Azz}{\dot{\theta }}_{E}\cos\theta_{E}\cos\theta_{R}+J_{Azz}{\dot{\theta }}_{R}\sin\theta_{E}\sin\theta_{R}\\ +{\dot{\theta }}_{E}\cos\theta_{E}\cos\theta_{R}-{\dot{\theta }}_{R}\sin\theta_{E}\sin\theta_{R}-K_{Af}\sin\theta_{E}\cos\theta_{R})\omega_{Ex}+(J_{Azz}\sin\theta_{R}){\dot{\omega }}_{Ey}\\ +(J_{Azz}{\dot{\theta }}_{R}\cos\theta_{R}-{\dot{\theta }}_{R}\cos\theta_{R}+K_{Af}\sin\theta_{R})\omega_{Ey}+(J_{Azz}\cos\theta_{E}\cos\theta_{R}){\dot{\omega }}_{Ez}\\ +(-J_{Azz}{\dot{\theta }}_{E}\sin\theta_{E}\cos\theta_{R}-J_{Azz}{\dot{\theta }}_{R}\cos\theta_{E}\sin\theta_{R}-{\dot{\theta }}_{E}\sin\theta_{E}\cos\theta_{R}\\ +{\dot{\theta }}_{R}\cos\theta_{E}\sin\theta_{R}+K_{Af}\cos\theta_{E}\cos\theta_{R})\omega_{Ez}+\sin\theta_{E}\cos\theta_{E}\sin\theta_{R}\omega_{Ex}^{2}\\ +\sin\theta_{E}\cos\theta_{E}\sin\theta_{R}\omega_{Ez}^{2}+(\cos\theta_{E}\cos\theta_{R})\omega_{Ex}\omega_{Ey}+(-\cos^{2}\theta_{E}\sin\theta_{R}\\ +\sin^{2}\theta_{E}\sin\theta_{R})\omega_{Ex}\omega_{Ez}+(\sin\theta_{E}\cos\theta_{R})\omega_{Ey}\omega_{Ez}-J_{Azz}{\ddot{\theta }}_{E}\sin\theta_{R}\\ -J_{Azz}{\dot{\theta }}_{E}{\dot{\theta }}_{R}\cos\theta_{R}+{\dot{\theta }}_{E}{\dot{\theta }}_{R}\cos\theta_{R}=T_{ADz}+T_{Adrive}-T_{Af0}+K_{Af}({\dot{\theta }}_{E}\sin\theta_{R}+\omega_{Bz}) \end{array}$$

Equations (9), (13) and (14) are the dynamic equations of the three-axis inertially stabilized platform. The input of the model is the motor driving torque of the azimuth frame, the pitch frame and the roll frame. The controlled variable of the system is the angular velocity of the pitching frame relative to the inertia space, while the disturbance comes from the angular velocity of the aircraft, the coupling and friction between the frames, etc.

However, because the equation is too complex and contains a large number of quadratic terms of controlled variables, it is very difficult to design the controller. Thus, we simplify the model in a way that is similar to linearizing the equilibrium point, ignoring the quadratic term. As a result, we can obtain the following:

(15) $$G\ddot{\psi }+C\dot{\psi }=T_{Drive}+T_{d}$$

where $\psi =\int_{0}^{t}\omega_{E}(\tau )d\tau ={\left[\begin{array}{ccc} \psi_{Ey} & \psi_{Ex} & \psi_{Ez} \end{array}\right]}^{T}$ represents the angle of pitch frame relative to inertia space. The other parameters are defined as follows:

(16) $$G=\left[\begin{array}{ccc} g_{11} & 0 & 0\\ 0 & g_{23} & g_{25}\\ g_{31} & g_{33} & g_{35} \end{array}\right],C=\left[\begin{array}{ccc} g_{12} & 0 & 0\\ 0 & g_{24} & g_{26}\\ g_{32} & g_{34} & g_{36} \end{array}\right],T_{Drive}=\left[\begin{array}{c} T_{Edrive}\\ T_{Rdrive}\\ T_{Adrive} \end{array}\right],T_{d}=\left[\begin{array}{c} T_{dy}\\ T_{dx}\\ T_{dz} \end{array}\right]$$

(17) $$\begin{array}{l} T_{dy}=-K_{Ef}[(\omega_{Bx}\sin\theta_{A}-\omega_{By}\cos\theta_{A})\cos\theta_{R}-(\omega_{Bz}+{\dot{\theta }}_{A})\sin\theta_{R}]-T_{Ef0}\\ T_{dx}=T_{RDx}-T_{Rf0}+K_{Rf}(\omega_{Bx}\cos\theta_{A}+\omega_{By}\sin\theta_{A})\\ T_{dz}=J_{Azz}{\ddot{\theta }}_{E}\sin\theta_{R}+(J_{Azz}-1){\dot{\theta }}_{E}{\dot{\theta }}_{R}\cos\theta_{R}+T_{ADz}-T_{Af0}+K_{Af}({\dot{\theta }}_{E}\sin\theta_{R}+\omega_{Bz}) \end{array}$$

(18) $$\begin{array}{l} g_{11}=J_{Eyy}\\ g_{12}=K_{Ef}\\ g_{23}=J_{Rxx}\cos\theta_{E}\\ g_{24}={\dot{\theta }}_{E}\sin\theta_{E}(J_{Rzz}-J_{Ryy}-1)+K_{Rf}\cos\theta_{E}\\ g_{25}=J_{Rxx}\sin\theta_{E}\\ g_{26}=J_{Rxx}{\dot{\theta }}_{E}\cos\theta_{E}-{\dot{\theta }}_{E}\cos\theta_{E}(J_{Rzz}-J_{Ryy})+K_{Rf}\sin\theta_{E}\\ g_{31}=J_{Azz}\sin\theta_{R}\\ g_{32}={\dot{\theta }}_{R}\cos\theta_{R}(J_{Azz}-1)+K_{Af}\sin\theta_{R}\\ g_{33}=-J_{Azz}\sin\theta_{E}\cos\theta_{R}\\ g_{34}=(1-J_{Azz}){\dot{\theta }}_{E}\cos\theta_{E}\cos\theta_{R}+(J_{Azz}-1){\dot{\theta }}_{R}\sin\theta_{E}\sin\theta_{R}-K_{Af}\sin\theta_{E}\cos\theta_{R}\\ g_{35}=J_{Azz}\cos\theta_{E}\cos\theta_{R}\\ g_{36}=(1-J_{Azz}){\dot{\theta }}_{R}\cos\theta_{E}\sin\theta_{R}-(J_{Azz}+1){\dot{\theta }}_{E}\sin\theta_{E}\cos\theta_{R}+K_{Af}\cos\theta_{E}\cos\theta_{R} \end{array}$$

## 3. Design of the MIMO Fuzzy Sliding Mode Controller

Define the position error vector of the pitch frame relative to the inertia space of the three-axis inertially stabilized platform:

(19) $$e=\psi -\psi_{d}$$

where $\psi_{d}=\left[\begin{array}{ccc} \psi_{dy} & \psi_{dx} & \psi_{dz} \end{array}\right]\in R^{3\times 1}$ is the desired position vector. Without loss of generality, suppose that $\psi_{d}$ is a second order continuous differentiable function.

Let the sliding mode function be:

(20) $$s=\dot{e}+ce$$

where c is a positive definite diagonal array:

(21) $$c=\left[\begin{array}{ccc} c_{1} & 0 & 0\\ 0 & c_{2} & 0\\ 0 & 0 & c_{3} \end{array}\right]$$

where $c_{1},c_{2},c_{3}>0$. Thus, according to Equations (19) and (20), we can obtain the following:

(22) $$\dot{\psi }={\dot{\psi }}_{d}+s-ce$$

and:

(23) $$\ddot{\psi }={\ddot{\psi }}_{d}+\dot{s}-c\dot{e}$$

After substituting Equations (22) and (23) into (15), the system model becomes:

(24) $$G\dot{s}=G(c\dot{e}-{\ddot{\psi }}_{d})-c({\dot{\psi }}_{d}+s-ce)+T_{Drive}+T_{d}$$

Design the three-axis inertially stabilized MIMO fuzzy sliding mode control law as follows:

(25) $$T_{Drive}=-(u_{o}+u_{c})$$

where $u_{o}=\left(a+\sigma \right)s$ is the output compensation control amount; $a=diag\left\{\begin{array}{ccc} a_{1} & a_{2} & a_{3} \end{array}\right\}$ is the real coefficient matrix, $a_{1},a_{2},a_{3}>0$; and $\sigma =diag\left\{\begin{array}{ccc} \sigma_{1} & \sigma_{2} & \sigma_{3} \end{array}\right\}$ is an adaptive compensation coefficient matrix. The second item in Equation (25) $u_{c}={\left[\begin{array}{ccc} u_{c1} & u_{c2} & u_{c3} \end{array}\right]}^{T}$ is the output of fuzzy control introduced for the coupling between the three frameworks of the platform.

The design of the coupled fuzzy control quantity is introduced below [26,27]. The typical five-level fuzzy segmentation, which includes NB, NM, ZE, PM and PB, is selected for the input and output of the fuzzy inference system. The fuzzy rules are as follows:

If $s_{i}$ is NB, then $u_{fi}$ is NB
If $s_{i}$ is NM, then $u_{fi}$ is NM
If $s_{i}$ is ZE, then $u_{fi}$ is ZE
If $s_{i}$ is PM, then $u_{fi}$ is PM
If $s_{i}$ is PB, then $u_{fi}$ is PB

where $s_{i},i=1,2,3$ is the row element of the sliding mode function s, that is $s={\left[\begin{array}{ccc} s_{1} & s_{2} & s_{3} \end{array}\right]}^{T}$. Furthermore, $u_{fi},i=1,2,3$ are the outputs of the fuzzy inference system.

**Figure 3:**
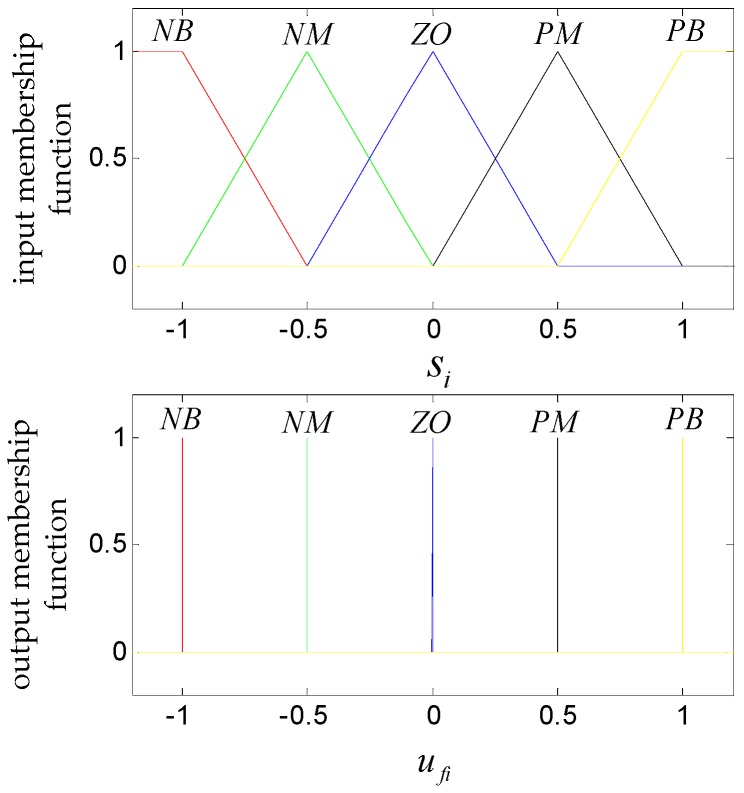
Input and output membership functions.

The selection of the membership function is empirically determined. The triangle membership function is selected as the input membership function and the single-value membership function is selected as the output membership function. The function curve is shown in Figure 3.

The defuzzification process of the fuzzy inference system is determined to be the weighted average method. The output of this method can be expressed as:

(26) $$u_{fi}=\frac{\sum_{R=1}^{5}\phi_{iR}\mu_{R}(s_{i})}{\sum_{R=1}^{5}\mu_{R}(s_{i})},\;\;\;i=1,2,3$$

where R=1,2,⋯,5 is the number of fuzzy rules; $\mu_{R}(s_{i})$ is the input membership function corresponding to the *R*-th rule; and $\phi_{iR}$ is the output membership function corresponding to the *R* -th rule. For the convenience of subsequent writing and discussion, we rewrite Equation (26) as:

(27) $$u_{fi}=\phi_{i}{}^{T}Y_{i},\;\;\;i=1,2,3$$

where $\phi_{i}={\left[\begin{array}{cccc} \phi_{i1} & \phi_{i2} & \phi_{i3} & \begin{array}{cc} \phi_{i4} & \phi_{i5} \end{array} \end{array}\right]}^{T}$, $Y_{i}={\left[\begin{array}{cccc} \begin{array}{cc} Y_{i1} & Y_{i2} \end{array} & Y_{i3} & Y_{i4} & Y_{i5} \end{array}\right]}^{T}$ and $Y_{i}=\frac{\mu_{R}(s_{i})}{\sum_{R=1}^{5}\mu_{R}(s_{i})},\;\;i=1,2,3$.

**Figure 4:**
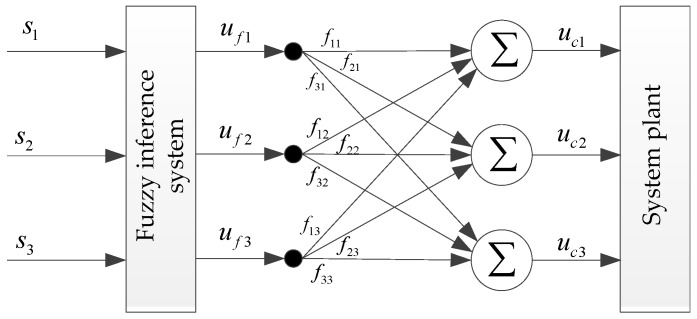
Structure diagram of fuzzy coupling compensation.

There is a serious coupling relationship between the frame axes of the three-axis inertially stabilized platform. In order to obtain the control effects with high precision, the coupling must be processed. In this paper, an adaptive fuzzy coupling compensation factor is adopted. The coupling compensation structure is shown in Figure 4 where $f_{ij}$ represents the coupling compensation factor of the *j*-th output to the *i*-th coupling compensation term $u_{ci}$ of the fuzzy inference system.

After this, the coupling compensation term $u_{c}$ in Equation (25) can be expressed as:

(28) $$u_{ci}=\sum_{j=1}^{3}f_{ij}u_{fj}=\sum_{j=1}^{3}f_{ij}u_{fj}\phi_{j}{}^{T}Y_{j},\;\;\;i=1,2,3$$

where the adaptive laws of $f_{ij}$ and $\sigma_{i}$ are:

(29) $${\dot{f}}_{ij}=\gamma_{ij}s_{i}u_{fj}$$

(30) $${\dot{\sigma }}_{i}=\eta_{i}s_{i}^{2}$$

and $\gamma_{ij},\eta_{i}>0,\;\;i,j=1,2,3$ is the proportional coefficient [26].

**Figure 5:**
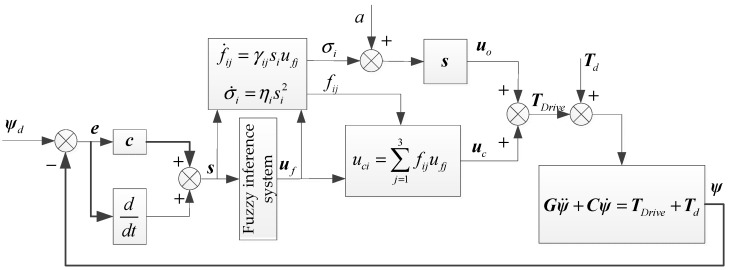
Structure of the MIMO fuzzy sliding mode control system with coupled adaptive compensation.

The structure of the MIMO fuzzy sliding mode control system with coupled adaptive compensation for the three-axis inertially stabilized platform is shown in Figure 5.

The stability and convergence of the three-axis inertially stabilized platform system described by Equation (15) under the action of control law (25), adaptive law (29) and (30) are demonstrated below [28].

Define the Lyapunov function as:

(31) $$V=s^{T}Gs+\frac{1}{2}\sum_{i=1}^{n}\sum_{j=1}^{n}\frac{1}{\gamma_{ij}}{\tilde{f}}_{ij}^{2}+\frac{1}{2}\sum_{i=1}^{n}\frac{1}{\eta_{i}}{\tilde{\sigma }}_{i}^{2}\;,n=3$$

where ${\tilde{f}}_{ij}$ is the coupling compensation factor estimation error and ${\tilde{\sigma }}_{i}$ is the output compensation factor estimation error.

Considering the first item on the right end of Equation (31), we define the following:

(32) $$V_{1}=s^{T}Gs$$

After this, we obtain:

(33) $$\begin{array}{ll} {\dot{V}}_{1} & ={\dot{s}}^{T}Gs+s^{T}\dot{G}s+s^{T}G\dot{s}\\ & =s^{T}(w{\dot{s}}^{T}Gs+\dot{G}s+G\dot{s})\\ & =s^{T}(B+T_{Drvie})\\ & =s^{T}\left[B-\left(a+\sigma \right)s-u_{c}\right] \end{array}$$

where $w={\left[\begin{array}{ccc} 1/3s_{1} & 1/3s_{2} & 1/3s_{3} \end{array}\right]}^{T}$ and $B=w{\dot{s}}^{T}Gs+\dot{G}s+G(c\dot{e}-{\ddot{\psi }}_{d})-c({\dot{\psi }}_{d}+s-c\dot{e})+T_{d}$. Thus, we can obtain:

(34) $$\begin{array}{ll} \dot{V} & ={\dot{V}}_{1}+\sum_{i=1}^{3}\sum_{j=1}^{3}\frac{1}{\gamma_{ij}}{\tilde{f}}_{ij}{\dot{\tilde{f}}}_{ij}+\frac{1}{2}\sum_{i=1}^{3}\frac{1}{\eta_{i}}{\tilde{\sigma }}_{i}{\dot{\tilde{\sigma }}}_{i}\\ & =\sum_{i=1}^{3}s_{i}(B_{i}-a_{i}s_{i}-\sigma_{i}s_{i}-u_{ci})+\sum_{i=1}^{3}\sum_{j=1}^{3}\frac{1}{\gamma_{ij}}{\tilde{f}}_{ij}{\dot{\tilde{f}}}_{ij}+\sum_{i=1}^{3}\frac{1}{\eta_{i}}{\tilde{\sigma }}_{i}{\dot{\tilde{\sigma }}}_{i} \end{array}$$

where $B_{i}$ is the row element of B. If we define $f_{ij}^{*}$ as the optimal estimated coupling compensation factor for B, there must be an optimal estimation error $\varepsilon_{i}>0$ that satisfies the following conditions:

(35) $$\left|B_{i}-\sum_{i=1}^{3}f_{ij}^{*}u_{fi}\right|\leq \varepsilon_{i}$$

Thus, ${\tilde{f}}_{ij}$ can be expressed as:

(36) $${\tilde{f}}_{ij}=f_{ij}-f_{ij}^{*}$$

Substituting Equation (36) into Equation (28), we can obtain the following:

(37) $$u_{ci}=\sum_{j=1}^{3}{\tilde{f}}_{ij}u_{fj}+\sum_{j=1}^{3}{\tilde{f}}_{ij}^{*}u_{fj},\;\;\;i=1,2,3$$

Define $\sigma_{i}^{*}\left|s_{i}\right|$ as the upper bound of $\varepsilon_{i}$, that is:

(38) $$\varepsilon_{i}\leq \sigma_{i}^{*}\left|s_{i}\right|,\;\;i=1,2,3$$

After this, ${\tilde{\sigma }}_{i}$ can be expressed as:

(39) $${\tilde{\sigma }}_{i}=\sigma_{i}-\sigma_{i}^{*}$$

After substituting Equations (37) and (39) into Equation (34), we obtain the following:

(40) $$\begin{array}{lll} \dot{V} & = & \sum_{i=1}^{3}s_{i}\left[B_{i}-a_{i}s_{i}-({\tilde{\sigma }}_{i}+\sigma_{i}^{*})s_{i}-\sum_{j=1}^{3}{\tilde{f}}_{ij}u_{fj}-\sum_{j=1}^{3}f^{*}{}_{ij}u_{fj}\right]\\ & & +\sum_{i=1}^{3}\sum_{j=1}^{3}\frac{1}{\gamma_{ij}}{\tilde{f}}_{ij}{\dot{\tilde{f}}}_{ij}+\sum_{i=1}^{3}\frac{1}{\eta_{i}}{\tilde{\sigma }}_{i}{\dot{\tilde{\sigma }}}_{i}\\ & = & -\sum_{i=1}^{3}a_{i}s_{i}^{2}+\sum_{i=1}^{3}\left[s_{i}\left(B_{i}-\sum_{j=1}^{3}f^{*}{}_{ij}u_{fj}\right)-s_{i}\sigma_{i}^{*}s_{i}\right]\\ & & +\sum_{i=1}^{3}\sum_{j=1}^{3}\left(\frac{1}{\gamma_{ij}}{\tilde{f}}_{ij}{\dot{\tilde{f}}}_{ij}-s_{i}{\tilde{f}}_{ij}u_{fj}\right)+\sum_{i=1}^{3}\left(\frac{1}{\eta_{i}}{\tilde{\sigma }}_{i}{\dot{\tilde{\sigma }}}_{i}-{\tilde{\sigma }}_{i}s_{i}^{2}\right) \end{array}$$

By combining Equations (35) and (38), we can obtain:

(41) $$s_{i}\left(B_{i}-\sum_{j=1}^{3}f^{*}{}_{ij}u_{fj}\right)\leq \left|s_{i}\right|\left|B_{i}-\sum_{j=1}^{3}f^{*}{}_{ij}u_{fj}\right|\leq \left|s_{i}\right|w_{i}$$

(42) $$\left|s_{i}\right|w_{i}\leq \sigma_{i}^{*}s_{i}^{2}$$

According to Equations (40)–(42), we can determine the following:

(43) $$\begin{array}{lll} \dot{V} & \leq & -\sum_{i=1}^{3}a_{i}s_{i}^{2}+\sum_{i=1}^{3}\left(\left|s_{i}\right|\varepsilon_{i}-s_{i}\sigma_{i}^{*}s_{i}\right)\;\\ & & +\sum_{i=1}^{3}\sum_{j=1}^{3}\left(\frac{1}{\gamma_{ij}}{\tilde{f}}_{ij}{\dot{\tilde{f}}}_{ij}-s_{i}{\tilde{f}}_{ij}u_{fj}\right)+\sum_{i=1}^{3}\left(\frac{1}{\eta_{i}}{\tilde{\sigma }}_{i}{\dot{\tilde{\sigma }}}_{i}-{\tilde{\sigma }}_{i}s_{i}^{2}\right)\\ & \leq & -\sum_{i=1}^{3}a_{i}s_{i}^{2}+\sum_{i=1}^{3}\sum_{j=1}^{3}\left(\frac{1}{\gamma_{ij}}{\tilde{f}}_{ij}{\dot{\tilde{f}}}_{ij}-s_{i}{\tilde{f}}_{ij}u_{fj}\right)+\sum_{i=1}^{3}\left(\frac{1}{\eta_{i}}{\tilde{\sigma }}_{i}{\dot{\tilde{\sigma }}}_{i}-{\tilde{\sigma }}_{i}s_{i}^{2}\right) \end{array}$$

By combining Equations (36) and (39) as well as the adaptive laws (29) and (30), we can obtain:

(44) $${\dot{\tilde{f}}}_{ij}={\dot{f}}_{ij}=\gamma_{ij}s_{i}u_{fj}$$

(45) $${\dot{\tilde{\sigma }}}_{i}={\dot{\sigma }}_{i}=\eta_{i}s_{i}^{2}$$

Therefore, Equation (43) becomes:

(46) $$\begin{array}{ll} \dot{V} & \leq -\sum_{i=1}^{3}a_{i}s_{i}^{2}\;+\sum_{i=1}^{3}\sum_{j=1}^{3}\left(\frac{1}{\gamma_{ij}}{\tilde{f}}_{ij}{\dot{\tilde{f}}}_{ij}-s_{i}{\tilde{f}}_{ij}u_{fj}\right)+\sum_{i=1}^{3}\left(\frac{1}{\eta_{i}}{\tilde{\sigma }}_{i}{\dot{\tilde{\sigma }}}_{i}-{\tilde{\sigma }}_{i}s_{i}^{2}\right)\\ & =-\sum_{i=1}^{3}a_{i}s_{i}^{2}+\sum_{i=1}^{3}\sum_{j=1}^{3}\left(\frac{1}{\gamma_{ij}}{\tilde{f}}_{ij}\gamma_{ij}s_{i}u_{fj}-s_{i}{\tilde{f}}_{ij}u_{fj}\right)+\sum_{i=1}^{3}\left(\frac{1}{\eta_{i}}{\tilde{\sigma }}_{i}\eta_{i}s_{i}^{2}-{\tilde{\sigma }}_{i}s_{i}^{2}\right)\\ & =-\sum_{i=1}^{3}a_{i}s_{i}^{2} \end{array}$$

As $a_{i}>0,i=1,2,3$, thus we obtain the following:

(47) $$\dot{V}\;\leq -\sum_{i=1}^{3}a_{i}s_{i}^{2}<0$$

According to the stability theory of Lyapunov, the stability and convergence are proved.

## 4. Experiments and Results

**Figure 6:**
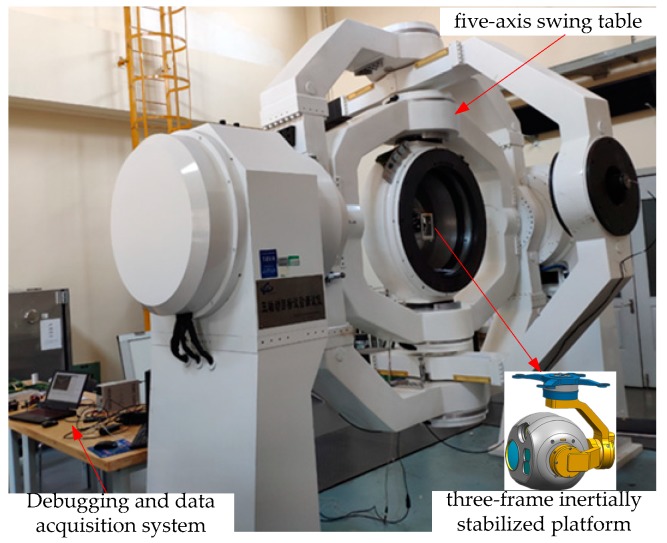
Experimental setup.

The experimental tests of the three-axis inertially stabilized platform are carried out in order to provide a comparison of the MIMO fuzzy sliding mode controller designed in this paper with the PI controller with each frame controlled separately. The experimental system is shown in Figure 6. The platform being tested is installed in the innermost frame of a five-axis swing table and the three-axis swing table inside the five-axis swing table is used to simulate the disturbance from the carrier. A three-axis gyroscope with high precision that is integrated in the innermost pitch frame of the three-axis inertially stabilized platform is used to measure the angular velocity of the system relative to the inertial space. An angle sensor is also provided on each frame axis with a resolution of $360/2^{19}$. The ground test system is used for debugging and data acquisition. Both of the tested controllers are implemented in DSP(TMS320F28335) using C language and the debugging software is CCS6.0.

In addition to testing the anti-disturbance performance of the system, the coupling between the three frameworks is emphatically tested. For example, when the azimuth frame tracks the input signal, the difference between the actual angular velocity and the input signal is the tracking error and the actual angular velocity of the pitch and roll frames is the coupling interference output. The carrier disturbance signal added in the experiment is calculated as follows: $\omega_{Bx}=\omega_{By}=\omega_{Bz}=2\pi \cos(2\pi t){(}^{\circ }/s)$.

We used the following parameters for the two controllers: (1) PI controller: Azimuth frame with $K_{AP}=8$ and $K_{AI}=20$; Roll frame with $K_{RP}=6$ and $K_{RI}=20$; Pitch frame with $K_{EP}=6$ and $K_{EI}=6$. (2) MIMO fuzzy sliding mode controller: $c_{1}=c_{2}=c_{3}=10$, $\eta_{1}=\eta_{2}=\eta_{3}=16$, $a_{1}=a_{2}=a_{3}=1$, $f_{ij}(0)=\sigma_{i}(0)=0$ and $\gamma =\left[\begin{array}{ccc} 10 & 1 & 1\\ 1 & 10 & 1\\ 1 & 1 & 10 \end{array}\right]$.

**Figure 7:**
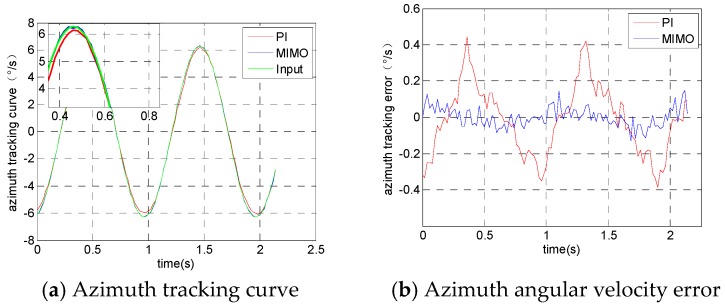

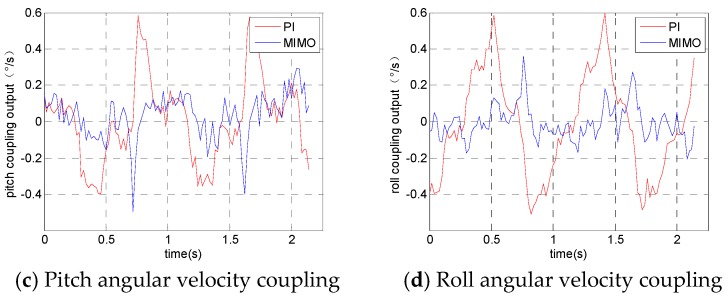
Angular velocity output when the azimuth frame tracks orders.

The experimental results from the azimuth frame tracking the input signal are shown in Figure 7. Figure 7a is the angular velocity tracking curve of the azimuth frame, Figure 7b is the azimuth frame tracking error curve, Figure 7c is the actual angular velocity output of the pitch frame and Figure 7d is the actual angular velocity output of the roll frame.

**Figure 8:**
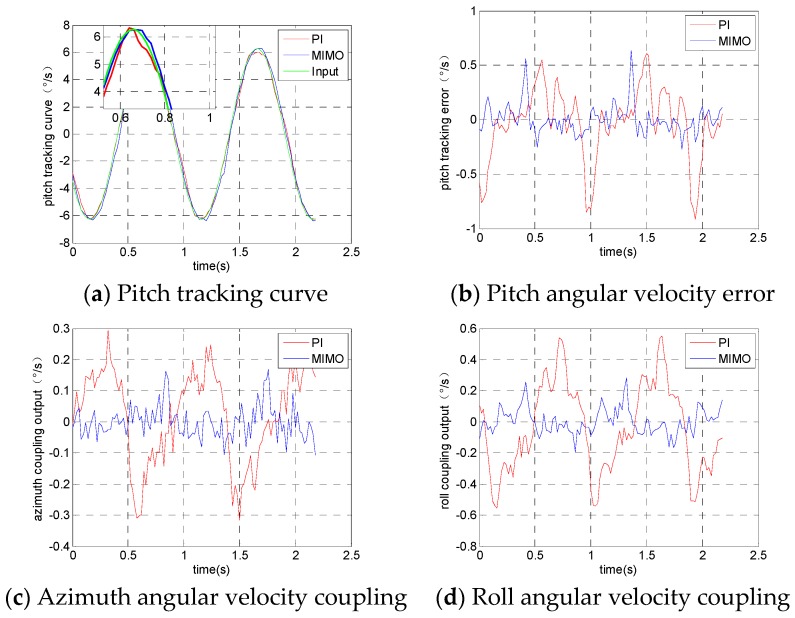
Angular velocity output when the pitch frame tracks orders.

The experimental results from the pitching frame tracking the input signal are shown in Figure 8. Figure 8a is the angular velocity tracking curve of the pitching frame, Figure 8b is the tracking error curve of the pitching frame, Figure 8c is the actual angular velocity output of the azimuth frame and Figure 8d is the actual angular velocity output of the rolling frame.

**Figure 9:**
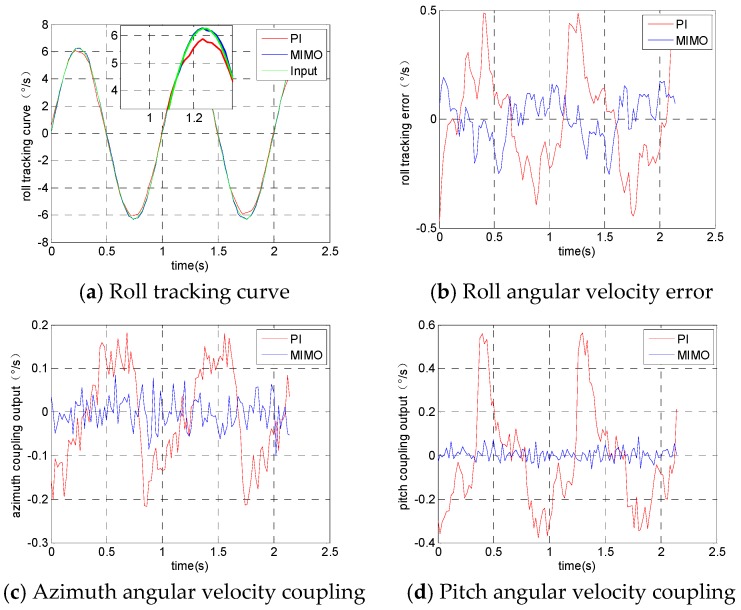
Angular velocity output when the roll frame tracks orders.

The experimental results from the rolling frame tracking the input signal are shown in Figure 9. Figure 9a is the angular velocity tracking curve of the rolling frame, Figure 9b is the tracking error curve of the rolling frame, Figure 9c is the actual angular velocity output of the azimuth frame and Figure 9d is the actual angular velocity output of the pitching frame.

It can be seen from the maximum value of the error curve and coupling output curve that the MIMO fuzzy sliding mode control method designed in this paper has advantages in terms of both the angular velocity tracking error of each frame and the coupling between them. In order to further investigate and quantitatively compare the experimental results, the integral absolute error (IAE) and the integral square error (ISE) are introduced [29]. ISE and IAE are defined as:

(48) $$IAE=\int_{0}^{T}\left|e(t)\right|dt$$

(49) $$ISE=\int_{0}^{T}{\left|e(t)\right|}^{2}dt$$

where e(t) is the measured error. The units of *IAE* and *ISE* are ${}^{{}^{\circ}}/s$ and ${{(}^{{}^{\circ}}/s)}^{2}$, respectively.

The results of the tracking error analysis are provided in Table 1, which indicates that the errors in the MIMO fuzzy sliding mode controller reach their minimum compared to those in the PI controller. This demonstrates the superiority of the proposed method in this paper.

The results of the coupling interference analysis are provided in Table 2. They indicate that the MIMO fuzzy sliding mode controller has stronger coupling suppression ability.

## 5. Conclusions

In this paper, the dynamic model of a three-axis inertially stabilized platform is established and its simplified model is given, in which the dynamic coupling of the three frames is fully considered. As the focus of this paper, a MIMO fuzzy sliding mode control method is designed to effectively resist internal and external disturbances. Furthermore, fuzzy logic is introduced to compensate for the coupling between frames. An experiment was designed to compare the PI controller with the controller proposed in this paper. The experimental results show that the MIMO fuzzy sliding mode control method has strong anti-disturbance ability and coupling suppression ability. The design and analysis method can be applied to the controller design of the three-axis inertially stabilized platform or other similar systems.

## Figures and Tables

**Table 1:** Tracking error analysis results.

	Azimuth Tracking Error		Pitch Tracking Error		Roll Tracking Error	
	PI	MIMO	PI	MIMO	PI	MIMO
**IAE**	0.04946	0.03487	0.08912	0.02613	0.02391	0.01886
**ISE**	0.0004632	0.00003871	0.0007954	0.00002871	0.0001934	0.0000845

**Table 2:** Coupling interference analysis results.

		Azimuth Coupling		Pitch Coupling		Roll Coupling	
		PI	MIMO	PI	MIMO	PI	MIMO
**Azimuth Tracking**	**IAE**	-	-	0.09745	0.03165	0.04237	0.00683
	**ISE**	-	-	0.0058	0.00069	0.00021	0.0000832
**Pitch Tracking**	**IAE**	0.07382	0.02509	-	-	0.02093	0.00482
	**ISE**	0.0043	0.00072	-	-	0.00013	0.0000317
**Roll Tracking**	**IAE**	0.03845	0.00541	0.04121	0.00692	-	-
	**ISE**	0.00018	0.0000613	0.00022	0.0000854	-	-
